# Synaptic Variability Introduces State-Dependent Modulation of Excitatory Spinal Cord Synapses

**DOI:** 10.1155/2015/512156

**Published:** 2015-06-11

**Authors:** David Parker

**Affiliations:** Department of Physiology, Development and Neuroscience, University of Cambridge, Cambridge CB2 3DY, UK

## Abstract

The relevance of neuronal and synaptic variability remains unclear. Cellular and synaptic plasticity and neuromodulation are also variable. This could reflect state-dependent effects caused by the variable initial cellular or synaptic properties or direct variability in plasticity-inducing mechanisms. This study has examined state-dependent influences on synaptic plasticity at connections between excitatory interneurons (EIN) and motor neurons in the lamprey spinal cord. State-dependent effects were examined by correlating initial synaptic properties with the substance P-mediated plasticity of low frequency-evoked EPSPs and the reduction of the EPSP depression over spike trains (metaplasticity). The low frequency EPSP potentiation reflected an interaction between the potentiation of NMDA responses and the release probability. The release probability introduced a variable state-dependent subtractive influence on the postsynaptic NMDA-dependent potentiation. The metaplasticity was also state-dependent: it was greater at connections with smaller available vesicle pools and high initial release probabilities. This was supported by the significant reduction in the number of connections showing metaplasticity when the release probability was reduced by high Mg^2+^ Ringer. Initial synaptic properties thus introduce state-dependent influences that affect the potential for plasticity. Understanding these conditions will be as important as understanding the subsequent changes.

## 1. Introduction

Variability is a feature of healthy physiological systems. Meanwhile, significant variability is recognized within neuronal and synaptic populations [1–11]; its relevance remains unclear [10, 11]. Plasticity effects are also variable [12–21]. This could reflect differences in plasticity-inducing mechanisms (e.g., second messenger pathways) or state-dependent effects caused by differences in initial cellular or synaptic properties [22].

This study has examined state-dependent influences on synaptic plasticity in the lamprey spinal cord by making paired recordings from connections made by glutamatergic excitatory interneurons (EINs) onto motor neurons. This connection exhibits marked variability and consists of functionally different subgroups [23]. The EINs provide the excitatory drive to the locomotor network [24] and are thus essential network components. Substance P presynaptically and postsynaptically modulates EIN inputs to motor neurons through changes in gene expression and synaptic ultrastructure [25–28]. Functionally, substance P potentiates low frequency-evoked EPSP amplitudes [29] and reduces depression during spike trains [28]. However, as with the basic properties of the connection [23], both forms of plasticity vary [25, 27–29].

The synaptic effects of substance P were initially examined on TTX-resistant miniature EPSPs, spontaneous EPSPs, and locomotor-related depolarizations [27]. These approaches are routinely used to examine synaptic interactions and modulation in spinal networks. However, they are indirect and do not identify changes at specific synapses and may not even reflect synaptic effects. Synaptic potentiation occurred in over 90% of these experiments [27, 30]. However, when monosynaptic EIN-evoked EPSPs were examined using paired recordings, potentiation occurred in 60–70% of experiments ([29]; see also [12, 17, 31]). This disparity could be caused by the activation of different types of neurons with different sensitivities to substance P when indirect approaches are used or that only a proportion of EINs is subject to modulation and significant effects are more likely when inputs are evoked simultaneously from multiple cells.

Small sample sizes prevented the variable plasticity of EINs inputs from being examined previously [25, 27, 28]. This study has used a larger sample of paired recordings to examine the influence of initial synaptic properties on plasticity. The results suggest that initial properties introduced state-dependent influences that affected the potential for synaptic plasticity.

## 2. Materials and Methods

Adult male and female lampreys (*Lampetra fluviatilis*) were obtained from commercial suppliers (Baitbox, Grimsby, UK). Animals were anaesthetized with MS-222 and the spinal cord and notochord were removed. A piece of spinal cord (1-2 cm) was isolated from the notochord and placed ventral side up in a Sylgard-lined chamber where it was superfused with Ringer containing (in mM): 138 NaCl, 2.1 KCl, 1.8 CaCl_2_, 1.2 MgCl_2_, 4 glucose, 2 HEPES, and 0.5 L-glutamine. The Ringer was bubbled with O_2_ and the pH adjusted to 7.4 with 1 M NaOH. The experimental chamber was kept at a temperature of 10–12°C.

Paired recordings were made from excitatory interneurons (EINs) and motor neurons using thin walled micropipettes filled with 3 M potassium acetate and 0.1 M potassium chloride to reduce tip potentials. Motor neurons were identified by recording orthodromic extracellular spikes in a ventral root following current injection into their somata. EINs were identified by their ability to elicit monosynaptic EPSPs in motor neurons (see [24]). Monosynaptic inputs were identified by their reliability and constant latency following presynaptic stimulation at 20 Hz [32]. While there is no evidence for regional differences in the EINs, to reduce potential location-dependent variability, experiments were only performed in the rostral trunk region just caudal to the last gill, and all EINs were recorded 1 segment rostral to the motor neuron. An Axoclamp 2A amplifier (Axon Instruments, California) was used for voltage recording and current injection. The motor neuron membrane potential was kept at −70 mV by injecting depolarizing or hyperpolarizing current using single electrode discontinuous current clamp (switching rate: 2 KHz). This was necessary to ensure that effects within and between experiments were not simply due to differences in membrane potential. While membrane potential differences could provide a physiologically relevant state-dependent variable, if it was not controlled across experiments, it would be impossible to separate synaptic variability from membrane potential variability [23]. The output of the sample-and-hold amplifier in the DCC circuit was monitored to ensure complete settling before voltage measurement. Data were acquired, stored, and analysed on computer using an analogue-to-digital interface (Digidata 1200, Axon Instruments, California) and Axon Instruments software (pCLAMP 8).

EIN action potentials were evoked by injecting 1 ms depolarizing current pulses of 0.5–3 nA. Low frequency-evoked EPSPs were examined by stimulating the EINs at 0.1 Hz. EPSPs were averaged (*n* = 10 sweeps) in control and after substance P application (1 *μ*M for 10 min; no activity-dependent plasticity occurs at this stimulation frequency). Effects over spike trains were examined by stimulating the EINs at 20 Hz for 1 s. The spike trains were evoked at 20 s intervals and averaged (*n* = 10) in control and after substance P application. Low frequency or train effects were not necessarily studied in all connections. Where spike trains were examined the initial EPSP in the spike train was used as a measure of the low frequency-evoked EPSP amplitude. While EIN stimulation at 20 Hz is a physiologically relevant frequency [33] over which substance P-evoked plasticity occurs [28], EINs only spike up to five times during fictive locomotion [34]. In contrast to previous analyses [25, 28], effects were only examined over the first five spikes to examine effects over the physiologically relevant range. The 1 s spike trains were used as they can be useful in providing insight into plasticity mechanisms (e.g., vesicle numbers; see below) while allowing effects over the physiological range to be selected.

EPSP amplitudes were measured as the peak amplitude above the baseline immediately preceding the presynaptic action potential. No attempt was made to correct for the effects of EPSP summation during spike trains [35] as there is usually little summation [28] and correcting for it did not significantly change measured EPSP amplitudes or their activity-dependent plasticity [23]. The initial EPSP amplitude and its change in substance P (EPSP_Subs  P_/EPSP_Control_), the paired pulse (PP) plasticity (EPSP_2_/EPSP_1_), and plasticity over the 2nd to 5th spikes in the trains (EPSP_Train_2–5__/EPSP_1_) were measured [25, 28].

**Figure 1:**
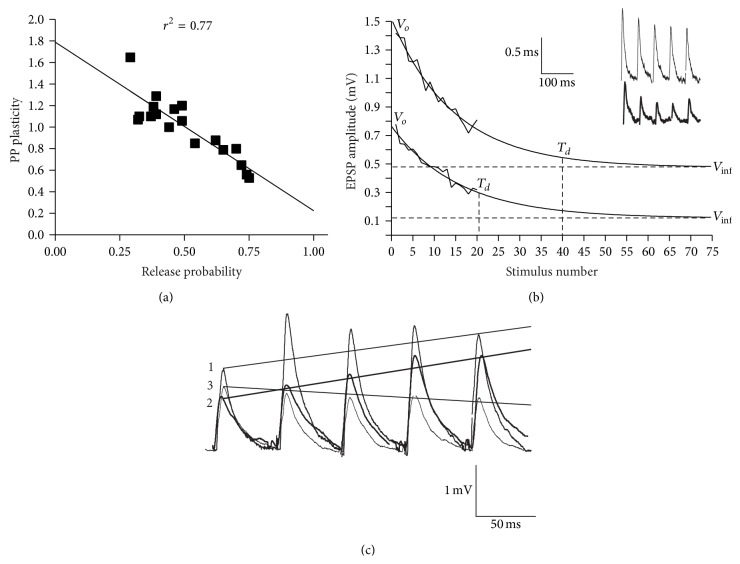
(a) Graph showing the significant relationship between the PP ratio and release probability using data from previous variance-mean analyses (see Section 2). (b) Analysis of the estimated available vesicle pool from the depression of connections. The inset shows two synapses that were depressed from different initial EPSP amplitudes. (c) Traces showing different forms of activity-dependent plasticity. Metaplasticity (1) is defined as reduced depression or increased facilitation from an increased initial EPSP amplitude; release probability-dependent facilitation (2; thick line) as facilitation from a reduced initial EPSP amplitude; and release probability-dependent depression (3) as depression from a larger initial EPSP amplitudes.

The release probability was determined directly in previous analyses using a variance-mean analysis [23, 25]. However, this requires relatively long-term recordings, which are rare [25]. The paired pulse (PP) ratio is routinely used to measure release probability, but the relationship between these effects is not simple as the PP ratio can be influenced by various presynaptic and postsynaptic effects [36–39]. Data from previous variance-mean analyses was used to examine the relationship between the PP ratio and the release probability ([23, 25]; Parker, unpublished data). There was a significant negative correlation (*r* ^2^ = 0.77, *n* = 18, *p* < 0.05; Figure 1(a)), which suggested that the PP ratio could be used to estimate the release probability.

The available synaptic vesicle pool was estimated from the depression of the EPSP over spike trains using the model of Wang and Zucker ([40]; see Figure 1(b)). This estimates the number of available vesicles from the initial and final PSP amplitude and the rate of depression (see Figure 1(b); [23]):

(1) $$\begin{array}{c} N_{\text{ves}}=\frac{V_{o}^{2}\tau d}{q\left(V_{o}-V_{\infty }\right)}, \end{array}$$

where *V*_*o*_ is the initial EPSP amplitude, *τd* is the inverse rate constant of EPSP decay (expressed as the number of presynaptic spikes needed for the EPSP to drop to 1/*e* of the initial value), *q* is the mean quantal amplitude (set at 0.1 mV; [23]), and *V*_*∞*_ is the EPSP amplitude at the plateau level of depression. However, not all connections depress [23], and in those that do depression to 1/*e* of the initial value often does not occur even over longer spike trains. This is probably due to the presence of simultaneous activity-dependent replenishment [41]. As a result, the extrapolated exponential depression calculated using the initial 2-3 EPSPs in the train was used to determine *τd* and *V*_*∞*_ to allow approximation of the vesicle pool [23]. Any replenishment would reduce the rate of rundown and thus cause overestimation of the initial vesicle pool.

Substance P (1 *μ*M) was applied once to each piece of spinal cord for 10 min. As it can evoke depolarizing oscillations and increased spontaneous synaptic inputs in motor neurons [30], synaptic inputs were only evoked after these effects had decayed (~2–5 min; [30]). *N* numbers in the text refer to the number of connections examined (only one synapse was examined in each piece of spinal cord). With the exception of the variance-mean data (Figure 1(a)), connections here were not used in previous analyses [25, 28, 29]. This was to ensure that all experiments were performed in the same species and under the same conditions. Stricter criteria for the synaptic changes were also used than in previous analyses [25, 28]. To be classed as potentiated or reduced, the low frequency-evoked EPSP amplitude had to be at least 110% or 90% of control, respectively, and for metaplasticity the initial EPSP had to be potentiated to at least 110% of control, and Train_2–5_ plasticity had to increase to at least 110% of control (1 on Figure 1(c)). These limits were imposed to focus on connections that showed relatively strong effects to help identify state-dependent changes associated with these forms of plasticity [10]. This resulted in the removal of 6 connections from the low frequency-evoked EPSP analysis and 10 from the spike train analysis. Statistical significance was examined using two-tailed paired or independent *t*-tests, Chi square, or one-way analysis of variance (ANOVA). When an ANOVA was used a Tukey test was used for post hoc analysis between groups. All values given refer to mean ± SEM.

## 3. Results

While they will be functionally related, for simplicity the influence of initial synaptic properties on the modulation of low frequency and train-evoked EPSPs will be presented separately.

### 3.1. Effects on Low Frequency-Evoked EPSPs

**Figure 2:**
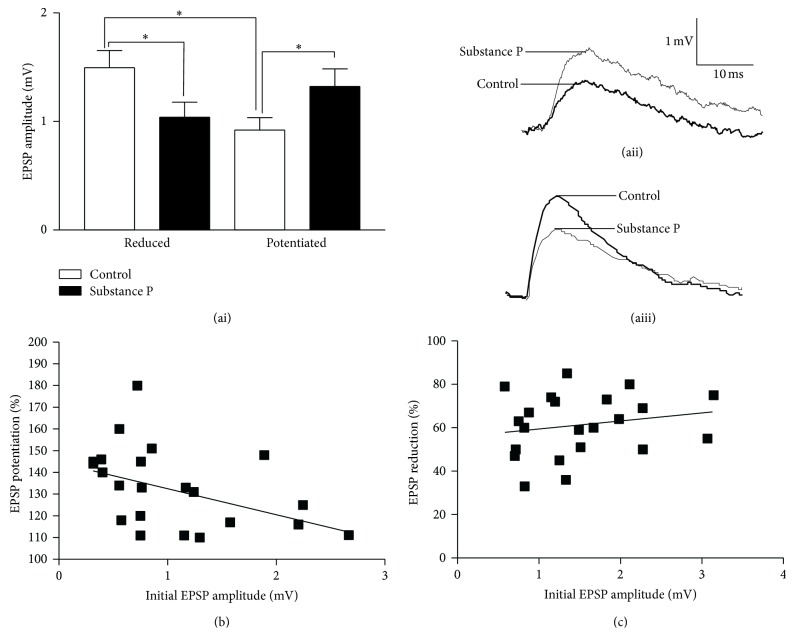
(ai) Graph showing the amplitude of the initial EPSP at connections that were potentiated or reduced by substance P. On this and other graphs, the asterisk indicates a statistically significant difference. Sample traces show connections where substance P potentiated (aii) or reduced (aiii) the initial EPSP amplitude. (b) Graph showing the significant negative relationship between the initial EPSP amplitude and the magnitude of the EPSP potentiation. (c) Graph showing the lack of a significant relationship between the initial EPSP amplitude and the magnitude of the EPSP reduction.

The substance P-mediated potentiation of low frequency-evoked EPSP amplitudes occurred in 22 of 44 connections (50%), a smaller proportion than that seen previously in a smaller sample size (70%; [29]). EIN-evoked EPSP amplitudes in motor neurons vary markedly (range 0.2 to ~4 mV; [23]). At potentiated connections, the amplitude of low frequency-evoked EPSPs was increased from 0.92 ± 0.11 mV to 1.32 ± 0.16 mV (*p* < 0.05; *n* = 22; Figure 2(ai)), and at connections where the EPSP amplitude was reduced, it was from 1.49 ± 0.15 mV to 1.04 ± 0.14 mV (*p* < 0.05, *n* = 22; Figures 2(ai)–2(aiii)). There was a significant difference in the amplitude of control EPSPs that were potentiated or reduced by substance P (*p* < 0.05; Figures 2(ai)–2(aiii)), suggesting that the initial EPSP amplitude influenced the direction of the modulation. There was also a significant negative correlation between the initial EPSP amplitude and the magnitude of the EPSP potentiation (*r* ^2^ = 0.2, *n* = 22, *p* < 0.05; Figure 2(b)) but no correlation between the initial EPSP amplitude and the magnitude of the EPSP reduction (*r* ^2^ = 0.04, *n* = 22, *p* > 0.05; Figure 2(c)). A similar asymmetric relationship between the initial EPSP amplitude and the direction of the synaptic modulation has been reported previously [12, 21, 42–45], but the underlying mechanisms are unknown.

**Figure 3:**
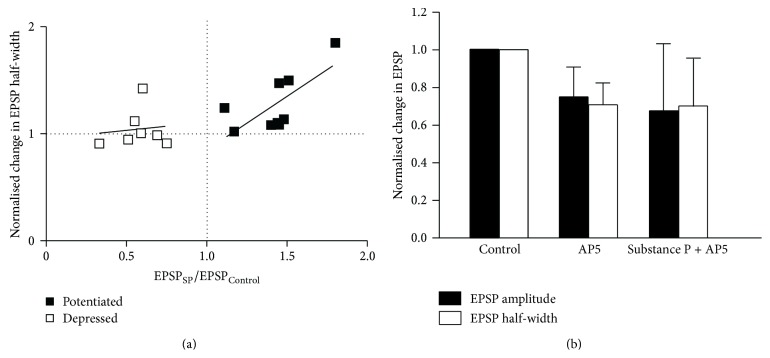
(a) Graph showing the relationship between the change in the EPSP amplitude (EPSP_Subs  P_/EPSP_Control_) and the EPSP half-width at connections that were reduced or potentiated by substance P. (b) Bar graph showing the effects of blocking the NMDA component of the EPSP with AP5 (100 *μ*M) on the EPSP modulation by substance P. Effects are shown in comparison to the normalized control EPSP amplitude and half-width.

The modulation of the EPSP amplitude could reflect presynaptic or postsynaptic effects (changes in quantal content or quantal amplitude; [46]). Substance P acts postsynaptically by potentiating the NMDA component of the EPSP (there is no evidence for AMPA-mediated effects; [47]). The role of NMDA receptor potentiation in the synaptic modulation was assessed using the EPSP half-width, which provides a measure of the NMDA component of the EPSP [48, 49]. Only connections in which the half-width could be clearly measured were used in the analysis. There was no significant difference in the control half-width at connections where the EPSP amplitude was potentiated or reduced by substance P (9.6 ± 1.2 ms (*n* = 9) compared to 9.9 ± 1.3 ms (*n* = 7), *p* > 0.05; data not shown), suggesting against a difference in the initial NMDA component of the EPSP. However, the half-width was not significantly altered at connections that were reduced by substance P (97 ± 7% of control; *p* > 0.05, *n* = 7; Figure 3(a)), but it was significantly increased at potentiated connections (127 ± 10%; *p* < 0.05, *n* = 9; Figure 3(a)). The potentiation also correlated with the magnitude of the half-width increase (*r* ^2^ = 0.50, *n* = 9, *p* < 0.05; Figure 3(a)), and thus the change in the EPSP amplitude scaled with the magnitude of the postsynaptic effect. If the postsynaptic NMDA-dependent effect influenced the EPSP potentiation, there should have been no potentiation when NMDA receptors were blocked. In AP5 (100 *μ*m) the EPSP amplitude and half-width were both reduced (data not shown). When substance P was applied in AP5, there was no significant effect overall on the EPSP amplitude or half-width (*n* = 8; Figure 3(b)). However, in individual connections, substance P could have no effect (*n* = 2), reduce (*n* = 5), or increase the EPSP amplitude (*n* = 1; data not shown), the variability suggesting the influence of other factors on the synaptic effects.

**Figure 4:**
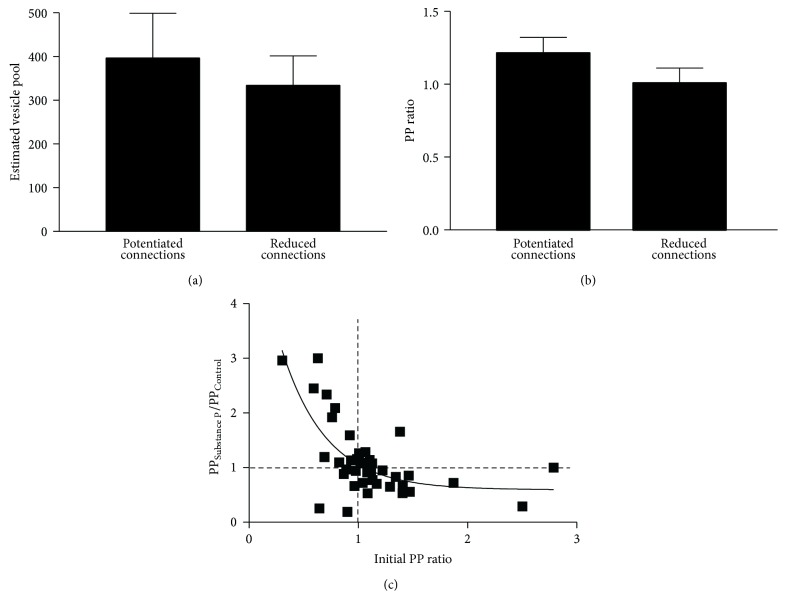
(a) Graph showing the size of the estimated available vesicle pool at connections that were potentiated or reduced by substance P. (b) Graph showing the PP ratio at connections that were potentiated or reduced by substance P. (c) Graph showing the relationship between the initial PP ratio and its change by substance P (PP_Subs  P_/PP_Control_).

Presynaptic influences on the low frequency-evoked EPSP modulation were examined by estimating the available vesicle pool size and the release probability (see Figure 1(b) and Section 2 for details). There was no significant difference in the size of the estimated vesicle pool at connections that were potentiated or reduced by substance P (396 ± 102 (*n* = 7) and 334 ± 67 (*n* = 11), resp.; *p* > 0.05; Figure 4(a)), suggesting against a difference in the initial vesicle pool size. The role of the initial release probability was determined from the paired pulse (PP) ratio of connections before substance P application. The initial PP ratio did not differ at connections that were potentiated or reduced (1.22 ± 0.1 (*n* = 22) compared to 1.01 ± 0.1 (*n* = 22); *p* > 0.05; Figure 4(b)). However, there was a significant exponential relationship between the initial PP ratio and its change in substance P (PP_Subs  P_/PP_Control_; *r* ^2^ = 0.47, *p* < 0.05; Figure 4(c)). While there are few points in the high initial PP ratio range, this relationship suggested that over a certain range there was a state-dependent influence of the initial release probability on its subsequent change. This occurred up to a PP ratio of approximately 1.5 (equivalent to a release probability of ~0.3; see Figure 1(a)). Where the PP_Subs  P_/PP_Control_ ratio was increased (i.e., the release probability was reduced by substance P; Figure 4(c)), it was greater when the initial PP ratio was low (i.e., a high initial release probability). In some connections there was a decrease in the PP_Subs  P_/PP_Control_ ratio. This indicated an increase in release probability by substance P, which could have contributed to the EPSP potentiation.

**Figure 5:**
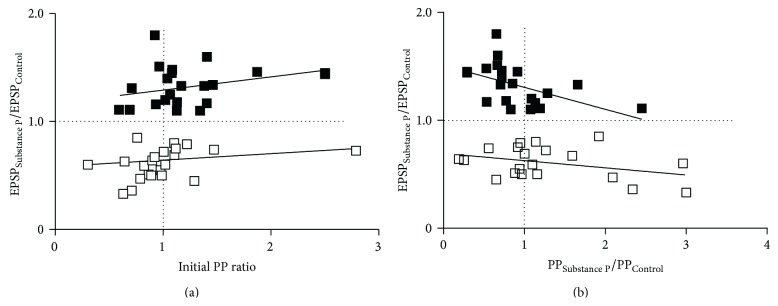
(a) The relationship between the initial PP ratio and the magnitude of the change in the EPSP amplitude by substance P (EPSP_Subs  P_/EPSP_Control_). Neither relationship was significant. (b) Graph showing the relationship between the change in the EPSP amplitude (EPSP_Subs  P_/EPSP_Control_) and the magnitude of the reduction in the PP ratio (PP_Subs  P_/PP_Control_). In this case, there was a significant negative correlation at potentiated connections.

The relationship between the EPSP amplitude and the PP ratio was examined by correlating the initial PP ratio to the change in the EPSP amplitude. When the EPSP amplitude was reduced (EPSP_Subs  P_/EPSP_Control_ < 1), there was no significant correlation between the extent of the EPSP reduction and the initial PP ratio (*r* ^2^ = 0.1, *n* = 22, *p* > 0.05; Figure 5(a)) or the change in the PP ratio by substance P (PP_Subs  P_/PP_Control_; *r* ^2^ = 0.13, *n* = 22, *p* > 0.05; Figure 5(b)). At connections potentiated by substance P (EPSP_Subs  P_/EPSP_Control_ > 1) there was again no significant correlation between the initial PP ratio and the change in the EPSP amplitude (EPSP_Subs  P_/EPSP_Control_; *r* ^2^ = 0.23, *n* = 10, *p* > 0.05; Figure 5(a)). However, there was a significant negative correlation between the PP_Subs  P_/PP_Control_ and the EPSP_Subs  P_/EPSP_Control_ (*r* ^2^ = 0.28, *n* = 22, *p* < 0.05; Figure 5(b)). A larger reduction of the release probability (i.e., PP_Subs  P_/PP_Control_ > 1) was thus associated with reduced potentiation. This may reflect a subtractive effect of the reduced presynaptic release probability on the postsynaptic potentiation of NMDA responses.

### 3.2. Substance P Effects during Spike Trains

Substance P can reduce depression or increase facilitation over spike trains [28]. This can occur with an increase or decrease of the initial EPSP amplitude to give metaplastic or release probability-dependent effects, respectively. Reduced depression or facilitation from an increased EPSP amplitude is defined as metaplasticity, while reduced depression form a smaller initial EPSP or increased depression from a larger initial EPSP is defined as release probability-dependent plasticity (RP_Fac_ and RP_Dep_, respectively; see Figure 1(c)). Metaplasticity occurred in 19 of 40 connections (48%, which is comparable to that in a smaller sample size, 58%; [25]), RP_Dep_ occurred in 7 connections, and RP_Fac_ occurred in 10 connections. In a small number of connections, substance P increased depression from a reduced initial EPSP amplitude (*n* = 4). This latter effect differs from that expected of a release probability-dependent plasticity mechanism and could be considered metaplastic depression: because of the small sample size these connections were not analysed further.

**Figure 6:**
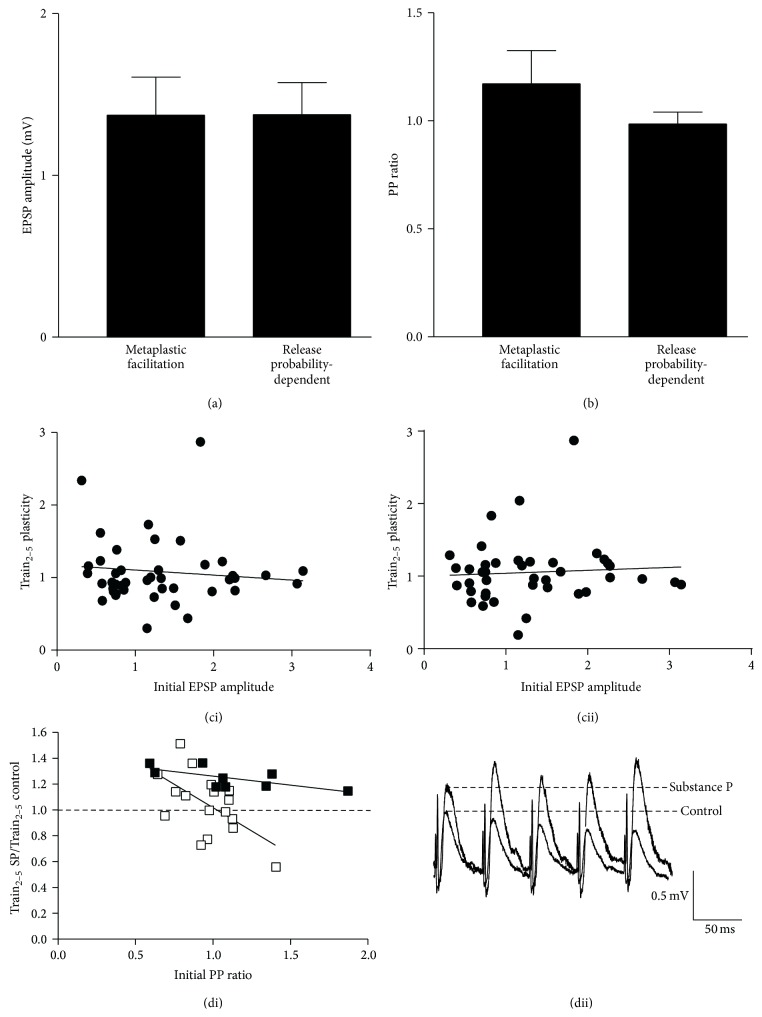
(a) Graph showing the lack of a significant difference in the EPSP amplitude at connections where metaplasticity or release probability-dependent plasticity occurred. (b) Graph showing the PP ratio at connections where different forms of activity-dependent plasticity occurred. (ci) Graph showing the relationship between the initial EPSP amplitude and the Train_2–5_ plasticity in control and in substance P (cii). (di) Graph showing changes in the PP ratio for the different forms of plasticity. (dii) Traces showing the changes in EPSP amplitude over spike trains.

Of the 19 connections that showed metaplasticity, 10 had a Train_2–5_ response after substance P that fell below the 110% cut-off limit (see Section 2), so only the 9 relatively strongly altered connections were analysed here. There was no significant difference in the control initial EPSP amplitude in the spike train (*p* > 0.05; Figure 6(a)) or the PP ratio (*p* > 0.05; Figure 6(b)) at connections where metaplasticity or release probability-dependent plasticity occurred. There was also no significant correlation between the initial EPSP amplitude and the activity-dependent plasticity in control (Train_2–5_ *r* ^2^ = 0.09; *p* > 0.05; Figure 6(ci)) or after substance P application (Train_2–5  Subs  P_ *r* ^2^ = 0.12, *p* > 0.05; Figure 6(cii)). The lack of correlation between the initial EPSP and the plasticity over spike trains presumably reflects the influence of multiple interacting parameters on synaptic properties (see [50, 51]).

The lack of a difference in the initial PP ratio suggested that the initial release probability did not determine the type of plasticity evoked. However, when the control PP ratio was related to the Train_2–5_ plasticity after substance P application (Train_Subs  P_/Train_Control_), there was a significant negative correlation between the initial PP ratio and the substance P-induced change in Train_2–5_ plasticity at connections where release probability-dependent plasticity (RP_Dep_ and RP_Fac_) or metaplasticity occurred (*r* ^2^ = 0.47 and 0.42, resp.; *p* < 0.05; see Figures 6(di) and 6(dii)). The reduced depression during spike trains, either metaplastic or release probability-dependent, was thus greater when the initial PP ratio was low (release probability was high).

**Figure 7:**
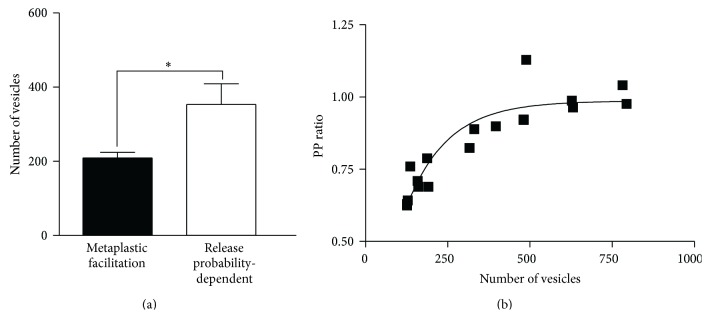
(a) There was a significant difference in the available vesicle pool at connections where metaplasticity or release probability-dependent plasticity occurred. (b) Graph showing the relationship between the number of vesicles and the PP ratio. The data is fit with a single exponential association and suggests that the influence of vesicle numbers on the PP ratio plateaued at approximately 1.0.

The number of vesicles in glutamatergic terminals is increased by substance P [25]. Given that there must be a physical limit on the number of vesicles at typical central synaptic terminals [52], a large initial vesicle pool could provide a state-dependent influence on the activity-dependent plasticity by limiting the potential for any increase. Support for this was obtained from estimates of the number of available vesicles before substance P application. This was significantly smaller at connections that showed metaplasticity (*n* = 4) than at those that exhibited release probability-dependent plasticity (*n* = 9; *p* < 0.05; Figure 7(a)).

While the quantal model assumes the vesicle pool and release probability are independent, they can be related. Larger vesicle pools are typically associated with higher release probabilities ([53–56]; but see [57]). The relationship between the vesicle pool size and the release probability was examined by relating the estimated number of available vesicles to the PP ratio. This relationship was fit by a single exponential association (*r* ^2^ = 0.83, *p* < 0.05, *n* = 16; Figure 7(b)), and thus over a certain range smaller estimated vesicle pools were associated with higher release probabilities (a similar effect occurs at the crayfish neuromuscular junction; [57]). This suggests a link between the smaller vesicle pools and high initial release probabilities that are needed to evoke metaplasticity.

### 3.3. Effects of Manipulating the Release Probability

While the analysis identified effects associated with the plasticity of EIN-evoked EPSPs, the analysis is correlational. An obvious approach is to alter the initial synaptic properties to put the synapses into functional states that should promote or inhibit the different forms of plasticity. However, this is complicated by the involvement of multiple presynaptic and postsynaptic effects that cannot be targeted specifically, either because their mechanisms are unknown or because specific tools are lacking (see [58]). The release probability was the obvious target as it had a state-dependent influence on both forms of plasticity and it can be manipulated (albeit not specifically). Attempts to change the state of the synapse were made here by reducing the release probability using high Mg^2+^ Ringer.

**Figure 8:**
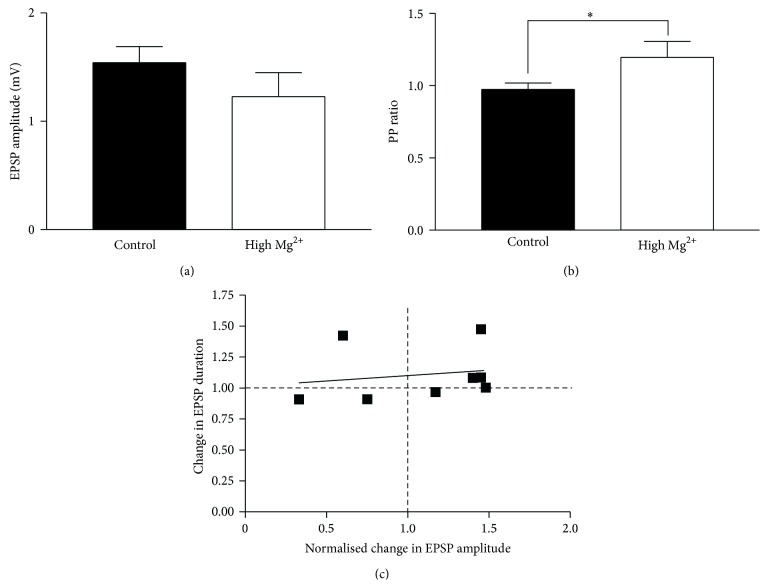
The effects of high Mg^2+^ Ringer on substance P-evoked synaptic modulation. Graphs show the lack of a significant effect of high Mg^2+^ on the EPSP amplitude (a) but a significant increase in the PP ratio (b), indicative of a reduction in release probability. (c) Graph showing the EPSP half-width at connections where the low frequency-evoked EPSP amplitude was potentiated or depressed by substance P.

High Mg^2+^ Ringer nonsignificantly reduced the initial EPSP amplitude (*n* = 20, *p* > 0.05) but significantly increased the PP ratio (*n* = 18, *p* < 0.05); (Figure 8(a)). The latter effect was consistent with a Mg^2+^-dependent reduction of the release probability. This should promote potentiation of the low frequency-evoked EPSP amplitude by substance P by reducing the potential subtractive effect of the reduction in release probability. However, in high Mg^2+^, 13 connections were potentiated (from 1.1 ± 0.3 mV to 1.5 ± 0.4 mV, *p* < 0.05), and 7 connections were reduced (from 1.4 ± 0.3 mV to 0.9 ± 0.3 mV, *p* < 0.05). The relative proportion of effects in high Mg^2+^ Ringer did not differ from that in normal Ringer (*p* > 0.5, Chi square), and thus reducing the release probability did not direct the plasticity of the synapses in the predicted way. A potential complication here is a change in the Mg^2+^ block of NMDA receptors [59, 60], which could reduce the postsynaptic potentiation and oppose the effects of the reduction in release probability on the potentiation. This was supported by analyses of the EPSP half width: in high Mg^2+^ Ringer there was no significant change in the EPSP half-width at connections that were either reduced or potentiated (*p* < 0.05, Figure 8(c)), suggesting a reduced postsynaptic effect.

While the opposing influences of high Mg^2+^ on the release probability and NMDA potentiation complicated attempts to direct the modulation of the initial EPSP, metaplasticity is not dependent on the postsynaptic NMDA effect [28], making this less of a confounding factor. The reduction in release probability in high Mg^2+^ Ringer (Figure 8(b)) should shift the synapse away from the region where metaplasticity would be promoted (Figure 6(di)). In high Mg^2+^ Ringer metaplasticity that exceeded the criteria used above did not occur in any connection (*n* = 16), although other effects occurred, including release probability-dependent depression (*n* = 6), release probability-dependent facilitation (*n* = 6), or increased depression from a lower initial EPSP amplitude (*n* = 2). Taking connections where changes below threshold occurred, metaplasticity still occurred in a significantly lower proportion of connections in high Mg^2+^ Ringer (*n* = 2 of 16) than in control Ringer (*n* = 19 of 40; *p* < 0.05 Chi square), consistent with metaplasticity being associated with relatively high initial release probabilities.

## 4. Discussion

In addition to characterizing the changes associated with synaptic and other forms of plasticity, given that plasticity is variable, there is also a need to focus on the conditions that influence its expression (see [61]). This study has extended the analysis of excitatory interneuron (EIN) synaptic properties [23, 62] to focus on how the marked variability of this connections influenced synaptic plasticity. The results suggest that variability of the synapse can influence the type and magnitude of synaptic modulation.

The potentiation of low frequency-evoked EPSPs reflected an antagonistic interaction between the postsynaptic NMDA-dependent potentiation of the EPSP and a state-dependent subtractive reduction of the release probability. Over spike trains, facilitation was the typical effect of substance P. This could occur with a reduction or increase in the initial EPSP amplitude (release probability-dependent plasticity and metaplasticity, resp.). State-dependent influences on whether metaplasticity rather than release probability-dependent plasticity was evoked were the initial release probability and the size of the available vesicle pool.

The changes in single EPSPs showed effects similar to soft bound plasticity considered in memory systems, where stronger synapses (larger EPSPs) potentiate less than weaker synapses, but a reduction of the EPSP is independent of the initial EPSP amplitude [12]. Bounds on potentiation may reflect resource addition (e.g., available vesicles), and as these are finite they place a limit on further potentiation. This may reflect a homeostatic mechanism that limits synapses to a range of values and so prevents potentiation above a certain level. However, as a reduction is subtractive, it could in principle occur from any level ultimately to zero. Activity-dependent changes, which determine synaptic strength over spike trains, showed no influence of the initial EPSP amplitude on the direction of the plasticity. The different influences of the initial EPSP amplitude on low frequency and activity-dependent synaptic plasticity suggest that making links between synaptic plasticity or modulation and the initial synaptic strength will depend on how the initial synaptic strength is defined (i.e., single inputs or effects over spike trains).

The changes in low frequency and activity-dependent effects over spike trains showed little correlation (see also [23]). While a state-dependent influence of the release probability was a common link, it had opposite influences: a high release probability favoured metaplasticity and a low release probability favoured potentiation of the initial EPSP amplitude. The lack of correlation between the initial and activity-dependent effects probably reflects the multiple factors that influence synaptic properties (e.g., [51]), with the net effect depending on the relative influence of different parameters. If activity-dependent synaptic depression was reduced by lowering the release probability, then the initial EPSP amplitude should also be reduced. However, with an associated postsynaptic potentiation of NMDA responses or an increase in vesicle numbers, a reduction of release probability can lead to reduced depression from an unchanged or increased initial EPSP. This offers greater flexibility than a single or fixed combined mechanism: release probability-dependent plasticity alone would redistribute effects over the spike train (depression occurring from larger initial EPSPs and facilitation from smaller EPSPs) and limit the potential for changes in overall synaptic strength, while synaptic scaling removes the potential computational advantages of activity-dependent plasticity over spike trains [63]. While low frequency and activity-dependent effects were separated here for analytical convenience, how they actually interact now requires analysis.

There was an interaction between the available vesicle pool and release probability, both of which influenced the metaplasticity. Larger vesicle pools are typically associated with larger release probabilities [53–56]. The opposite interaction occurred here where there was an inverse relationship between the release probability and the estimated vesicle pool size (see also [57]). The mechanisms underlying this interaction are unknown, but they could relate to increased vesicle competition for calcium or greater calcium buffering with high vesicle densities. The effect plateaued at a PP ratio of approximately 1, equivalent to a release probability of ~0.5. Matching a high release probability with a smaller vesicle pool offers the potential to increase vesicle numbers when there was a larger relative reduction in release probability. This could increase the quantal content despite a reduction in release probability allowing metaplasticity rather than the expected release probability-dependent facilitation [25]. This was supported by release probability-dependent facilitation at connections where the initial vesicle pool was larger.

While the effects selected for analysis here can account for some of the variability of plasticity, they clearly cannot account for it all (e.g., metaplasticity could fail at connections with small depressing EPSPs that suggest high initial release probabilities and small vesicle pools which offer the ideal initial conditions for inducing metaplasticity). This may reflect differences in induction mechanisms (e.g., activation of second messenger pathways; [64]) needed to trigger effects [47], random effects on synaptic processes [10], or as yet unidentified, state-dependent influences.

In addition to the selective analysis of low frequency and spike train effects, an additional weakness is that it is largely correlative. This reflects the general difficulties of examining and altering synaptic properties. Attempts at directing plasticity by altering the release probability in high Mg^2+^ Ringer were not ideal as NMDA properties would also be affected. Altering Ringer Ca^2+^ levels was not an option as it would affect many other calcium-dependent presynaptic [40, 65] and postsynaptic processes (changes in NMDA receptor conductance and desensitization; [66]). However, for the metaplasticity (where NMDA-dependent effects do not appear to be necessary; [28]), there was support for the state-dependent influence of the initial release probability.

### 4.1. Functional Implications

Variability is necessary to any adaptive system [67, 68]. EIN cellular and synaptic properties vary markedly [23, 34, 69]. Assuming that this is not random variation in EIN properties, this suggests the presence of functional subpopulations [23]. As the EINs provide the excitatory drive to the network [24], the activation of different subpopulations should alter the motor output. This could potentially account for the variability of fictive locomotion (see [47, 70]). While it has seldom been discussed (see [71]), the variable output evoked under what seems to be identical conditions is arguably a defining feature of fictive locomotor activity. This variability could be explained by the random activation of different populations of EINs by bath applied glutamate agonists.

The network relevance of variable cellular and synaptic properties is uncertain [11] and remains to be determined in spinal cord locomotor networks. The network effects of substance P are also variable and state-dependent: the modulation of the frequency and regularity of network activity both vary markedly [47], and both depend on their initial values [47], although it is not known how this relates to the state-dependent effects on single synapses suggested here. Pérez et al. [72] show a disparity in substance P effects as their increase in burst frequency was less than a previous analysis [47]. This may reflect the problems of getting stable fictive activity, but the time that preparations were left to reach stability (4 h in [47]) matches the time needed for stability in [72]. However, the large effects Pérez et al. compare to only occur from low initial frequencies [47]; the disparity largely disappears when similar starting frequencies are considered (e.g., a starting frequency of 1.5 Hz gives ~20% increase in [72] compared to ~40% in [47]). Nevertheless, arguably the most constant feature of fictive activity is its variability, which obviously complicates studies of network mechanisms. Ideally, the initial state of synapses would be altered to see if network modulation could be directed, but this would be difficult due to the multiple cellular and synaptic effects involved in network function and plasticity.

Substance P can modulate variability [62], an effect also seen here. In the sample size examined, in control, 47% of connections depressed, 37% were facilitated, and 16% were unchanged over Train_2–5_ (this approximately matched the proportions in a much larger sample where 46% depressed, 27% were facilitated, and 12% were unchanged; [23]). After substance P application 29 of 40 connections were facilitated (72.4%; this is either release probability-dependent or metaplastic). Substance P thus reduced the variability of activity-dependent plasticity. The effect that this has on the network can be considered as the relative excitatory drive to motor neurons calculated as

(2) EPSPInit×Train2–5  Plasticity×Proportion of connections.

In control, *D* = 0.43, *F* = 0.66, and *U* = 0.32, giving a total of 1.41 in control and 1.34 after substance P. This argues against the assumption that substance P alters the network output by increasing network excitability by increasing glutamatergic drive [47] and instead suggests a change in EIN synaptic properties that preserve their overall synaptic effect. However, substance P also increases EIN excitability and reduces spike variability [27, 62], effects that could lead to enhanced activation and synchronization of the EIN pool.

## 5. Conclusions

This analysis adds to the evidence for significant synaptic variability in spinal locomotor networks [73–79]. The EINs have been referred to as a “relatively homogeneous” population [80], a claim that ignored the known variability of these cells and their synaptic connections [23, 34, 62, 69]. It has also been stated that there has been no analysis of different functional classes of the EINs [81], a claim that ignores the analysis of functional subdivisions within this interneuron population [23]. This variability can be modulated [62], and, as shown here, the variability can influence the potential for synaptic plasticity. In addition to determining the functional roles of different cell classes in spinal cord (and other) networks, the variability suggests that we will have to consider finer subdivisions in cell populations than suggested by traditional anatomical, molecular, or physiological markers. In lamprey and other systems, network synaptic interactions are routinely examined from spontaneous or locomotor-related PSPs or inputs evoked by extracellular stimulation. While these approaches have the advantage of speed, they essentially average across an unknown number of unknown inputs and ignore the heterogeneity of cellular and synaptic populations. Given the increasing awareness of the importance of variable parameters in network function (see [82]), the limitations of these approaches to understanding network function and plasticity need to be acknowledged and addressed.
